# Sources of Frustration Among Patients Diagnosed With Renal Cell Carcinoma

**DOI:** 10.3389/fonc.2019.00011

**Published:** 2019-01-22

**Authors:** Cristiane Decat Bergerot, Dena Battle, Paulo Gustavo Bergerot, Nazli Dizman, Eric Jonasch, Hans J. Hammers, Daniel J. George, Axel Bex, Borje Ljungberg, Sumanta Kumar Pal, Michael D. Staehler

**Affiliations:** ^1^Department of Medical Oncology and Experimental Therapeutics, City of Hope Comprehensive Cancer Center, Duarte, CA, United States; ^2^Kidney Cancer Research Alliance (KCCure), Alexandria, VA, United States; ^3^University of Texas MD Anderson Cancer Center, Houston, TX, United States; ^4^University of Texas Southwestern Medical Cancer Center, Dallas, TX, United States; ^5^Duke University Medical Center, Duke Cancer Institute, Durham, NC, United States; ^6^Division of Surgical Oncology, Department of Urology, Netherlands Cancer Center, Amsterdam, Netherlands; ^7^Department of Surgical and Perioperative Sciences, Urology and Andrology, Umeå University, Umeå, Sweden; ^8^Department of Urology, Ludwig-Maximilians University, Munich, Germany

**Keywords:** renal cell carcinoma, health care survey, frustration, fear of cancer recurrence, qualitative study

## Abstract

Despite numerous therapeutic advances in renal cell carcinoma (RCC), little is known about patients' perspectives on cancer care. An international survey was conducted to identify points of frustration associated with cancer care reported by patients with RCC. Data were obtained from an online survey, conducted from April 1 to June 15, 2017, through social media and patient networking platforms. This survey obtained baseline demographic, clinicopathologic, and treatment-related information. Open-ended questions accessed sources of frustration in cancer-related care and patients' suggestions for amelioration. Responses were categorized and reviewed by independent reviewers. A qualitative analysis was performed and the Kruskal-Wallis test was used to define associations between baseline characteristics and sources of frustration. Among 450 patients surveyed, 71.5% reported sources of frustration, classified as either emotional (48.4%) or practical (23.1%). The most common were fear of recurrence/progression (15.8%), distrust of their cancer care system (12.9%), and lack of appropriate information (9.8%). Female gender and non-clear cell histology were associated with both types of frustration, and older age was linked to practical sources of frustration. Patients suggested solutions included greater compassion among health care practitioners (20.7%), better access to information (15.1%) and research to improve their chances of being cured (14.7%). Sources of frustration related to emotional and practical causes were identified amongst patients with RCC. Certain demographic and clinical characteristics were associated with more sources of frustration. This study provides the first characterization of specific ways to improve the patient experience by addressing common frustrations.

## Introduction

The management of metastatic renal cell carcinoma (mRCC) is undergoing a rapid change. Vascular endothelial growth factor (VEGF)-directed therapies supplanted cytokine therapies a decade ago (1–3). Shortly thereafter, inhibitors of the mammalian target of rapamycin (mTOR) were introduced (4). Most recently, checkpoint inhibitors (directed at programmed death-1 [PD-1] and common tumor leukocyte antigen 4 [CTLA4]) have shown benefit in mRCC, and represent a first line standard of care for RCC (5, 6). Combinations of these agents have shown substantial promise and will likely soon represent the first-line standard of care (7). Historically, for the patient with localized disease, treatment options have been relatively straightforward (i.e., surgery or other local definitive options). Now, with the FDA approval of adjuvant the VEGF-tyrosine kinase inhibitor (VEGF-TKI) sunitinib, adjuvant systemic therapy is also a potential consideration for the postoperative patient (8).

It has been increasingly challenging for medical oncologists and urologists to keep pace with the rapidly changing environment of available therapies. One must assume that this represents an even greater issue from the perspective of the patient. In the current study, we queried patients with RCC regarding points of frustrations associated with medical care. Our questions were open-ended, allowing for a diverse array of responses. To our knowledge, this represents the first effort to pinpoint sources of frustration in RCC care from a patient's perspective.

## Materials and Methods

### Survey Development and Distribution

A panel was assembled comprised of members of the European Association of Urology (EAU) and the Kidney Cancer Research Alliance (KCCure), a US-based non-profit patient advocacy program. The panel had two aims in generating a patient survey: (1) to ascertain perspectives and attitudes of RCC patients toward adjuvant systemic therapy, and (2) to identify points of frustration related to their care. Ultimately, a 12 question survey was generated. Baseline demographic information was obtained including gender, age, and race. Diagnosis and treatment related data was also collected, including date of diagnosis with RCC, presence or absence of nephrectomy, stage at diagnosis, histologic subtype and presence or absence of recurrence (for patients with localized disease). Questions related to adjuvant therapy perspectives are listed in Supplemental Table 1; responses to these questions have been reported separately (9). The panel generated two open-ended questions to ascertain points of frustration in medical care, as follows:

- In your own words, what has frustrated you most about your medical care related to your diagnosis?
- If you could improve something about the medical care system for future patients diagnosed with kidney cancer, what would it be?

The survey was hosted on KCCure's website, and distributed via surveymonkey® through international patients forums using social media (“Facebook”; http://www.facebook.com) and patient-facing blog (“Smart Patients”; http://www.smartpatients.com) addressing approximately 800 patients from April 1, 2017 to June 15, 2017. Duplicate responses were eliminated before the data were analyzed. Responses to the survey were only considered for patients who noted a diagnosis of RCC; responses from practitioners or patients with non-RCC diagnoses were not considered.

### Characterization of Open-Ended Responses

To offer a semi-qualitative assessment of open-ended responses, we characterized patient frustrations into two broad categories, either practical or emotional. Practical frustrations were characterized as those centered around (1) financial issues, (2) lack of appropriate information, (3) lack of communication within care team, and (4) supportive care. Emotional frustrations were characterized as those centered around (1) fear of recurrence/progression, (2) distrust of cancer care system, (3) communication between patient and physician, (4) side effects, (5) lack of available/relevant research, and (6) mistrust of physician's knowledge. To illustrate the nature of responses by category, several examples are included in Table 1.

**Table 1:** Sources of frustration cited by survey respondents with RCC with semi-qualitative characterization of responses.

**Source of frustration theme**	**Representative quotation**	**Frequency(%)**
**EMOTIONAL**		
Fear of recurrence or progression	“I just have to sit and wait and hope it doesn't return”	15.8
Distrust of cancer care system	“My tumor was misdiagnosed as a cyst and then completely missed in scan 2 years ago”	12.9
Communication between patient and physician	“I don't think my doctor really listens or takes me seriously when I talk about the pain in the area of my surgery”	8.7
Lack of available/relevant research	“Lack of research on RCC and on treatment”	3.8
Mistrust of physician's knowledge	“Doctors not knowing enough about kidney cancer”	3.8
Side effects	“Too many new problems after resolution (i.e., chronic pain and fatigue)”	3.1
**PRACTICAL**		
Lack of appropriate information	“Lack of knowledge and treatment options for my type of cancer”	9.8
Financial	“Insurance regulating what they think I need and not may cancer specialist”	8.7
Supportive care	“Mental health not really considered. Little support groups”	2.7
Lack of communication within care team	“Lack of coordination between doctors for additional health issues”	2.4

Survey respondents were also offered an opportunity to provide (also in an open-ended fashion) suggestions for how they might resolve their cited frustrations. The resulting responses were also characterized in a semi-qualitative fashion, using categories cited in Table 2.

**Table 2:** Solutions proposed by survey respondents with RCC, with semi-qualitative characterization of responses.

**Improvement theme**	**Representative quotation**	**Frequency(%)**
Physicians and compassion	“Spend more time listening to patients”	20.7
More information	“Clear understanding of all treatment option from reliable sources” “Receive a treatment roadmap and a better understanding of Fuhrman grade”	15.1
Research	“Develop a test to detect RCC earlier” “We need to find a cure so we can all know there is an end to our treatments and maybe feel normal again”	14.7
Less financial issues	“Reasonable costs for medications” “Find a way to make medical care accessible & affordable to all who need it”	11.1
Supportive care	“Newly diagnosed need a social worker for guidance” and “Whole body care”	5.6
Better communication	“More agreement among doctors”	4.7
Symptom management	“Better pain management, and the long term effects this disease has on your body over time”	1.8

### Statistical Analysis

We performed a qualitative content analysis, as proposed by Bardin (10). Each response was analyzed by 2 independent reviewers (CB and PB) and categorized into one of several descriptive categories. Discrepancies were discussed and adjudicated by consensus, with an almost perfect agreement of 0.85, tested with Cohen's kappa coefficient. The Kruskal-Wallis test was used to define associations between the baseline characteristics and sources of frustration, as characterized by the previously noted algorithm.

## Results

### Patient Characteristics

Of 800 respondents to the survey, 450 met eligibility for our current analysis (e.g., non-duplicate responses originating from patients with an RCC diagnosis). Amongst patients with RCC, median age was 55.6, with 56.4% females and 43.6% males. RCC patients were primarily white (92.9%) and 116 patients (25.8%) were stage IV at the time of diagnosis (Table 3). The most frequent histologic subtype was clear cell (76.4%), followed by papillary (3.6%) and chromophobe (3.6%). Other non-clear cell histologies included translocation RCC (2.0%), unclassified (3.8%) and collecting duct (0.7%)—histologic subset was unknown in 9.8% of patients. Amongst those patients that had non-metastatic disease at the time of diagnosis, 39.2% were noted to be disease-free at the time of the survey.

**Table 3:** Demographic and clinical characteristics (*N* = 450).

**Characteristics**	**M(Range)/N(%)**
Age (Median, Range)	55.6 (17–82)
**GENDER**	
Female	254 (56.4)
Male	196 (43.6)
**RACE**	
White	418 (92.9)
Hispanic	13 (2.9)
Asian	9 (2.0)
Black	2 (0.4)
Other	8 (1.8)
**HISTOLOGY**	
Clear cell	344 (76.4)
Papillary	16 (3.6)
Chromophobe	16 (3.6)
Translocation xp11.2	9 (2.0)
Collecting duct	3 (0.7)
Unclassified	17 (3.8)
Unknown	44 (9.8)
**DISEASE STAGE**	
I	133 (29.6)
II	86 (19.1)
III	101 (22.4)
IV	116 (25.8)
Unknown	14 (3.1%)
Prior nephrectomy	330 (73.3)
Presence of recurrence^*^	274 (60.8)

* *Note that presence of recurrence is salient to those patients who were initially noted to have localized disease*.

### Sources of Frustration

Among patients with RCC, 71.5% of patients noted having some source of frustration in their care. Emotional causes of frustration were more common than practical causes of frustrations (48.4 vs. 23.1%; *P* = 0.001), as shown in Table 1. Amongst emotional causes of frustration, the most common causes were fear of recurrence or progression (15.8%), distrust of cancer care system (12.9%) and communication between patient and physician (8.7%). Amongst practical causes of frustration, the most common drivers were lack of appropriate information (9.8%), financial (8.7%) and lack of access of supportive care (2.7%).

### Association of Frustration With Patient Characteristics

**Figure 1:**
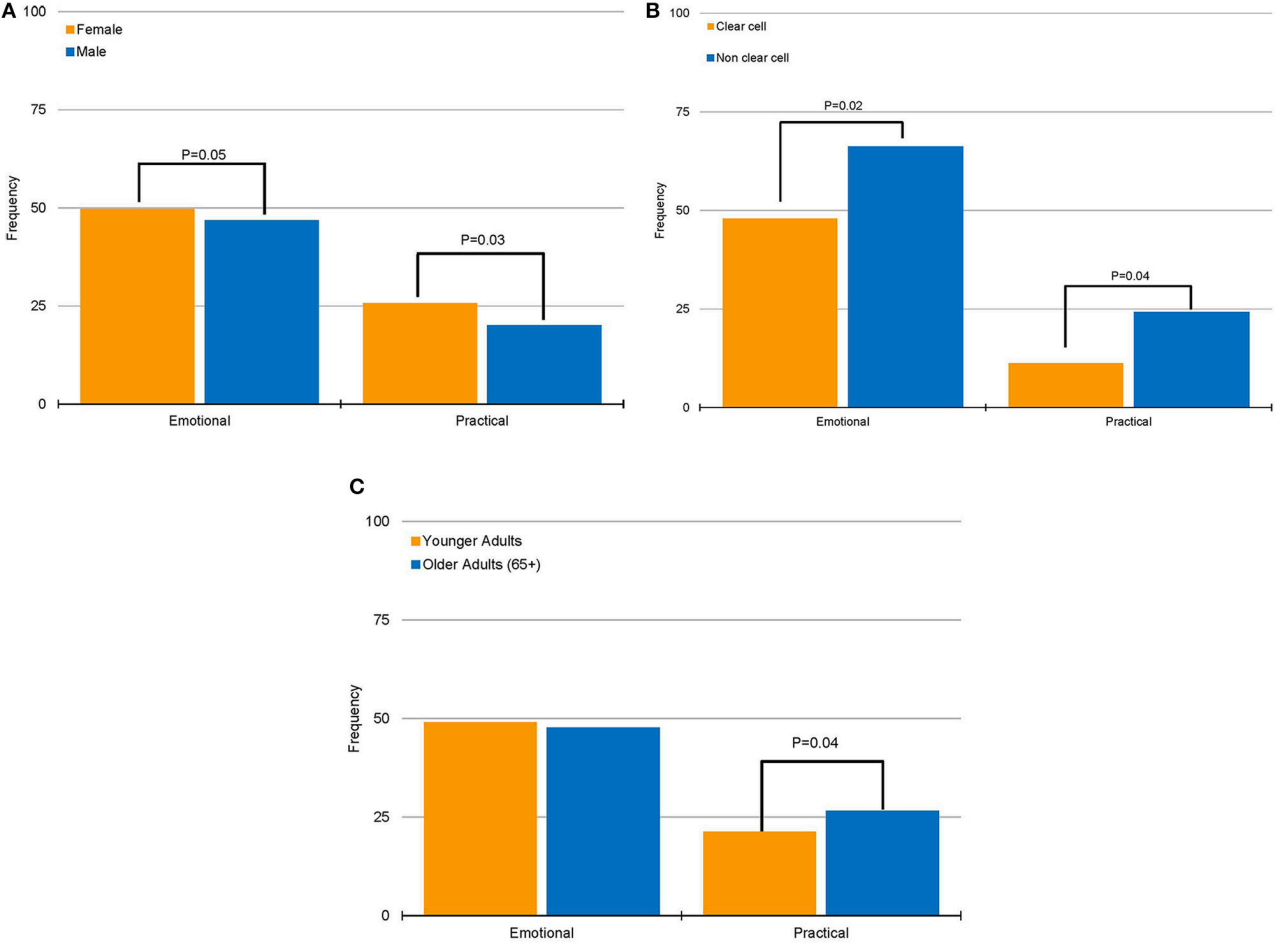
Associations of emotional and practical sources of frustrations with patient characteristics. **(A)** Association between gender and sources of frustration. **(B)** Association between histology group and sources of frustration. **(C)** Association between age group and sources of frustration.

When assessing sources of frustration dichotomized by baseline characteristics, we identified that females and patients with non-clear cell histology more frequently reported practical (*P* = 0.03 and *P* = 0.04, respectively; Figures 1A,B) and emotional (*P* = 0.05 and *P* = 0.02, respectively; Figures 1A,B) sources of frustration. The most common types of frustration encountered by females were fear of recurrence/progression (17.7%), distrust of cancer care system (15.0%), and lack of appropriate information (10.6%). Among patients with non-clear cell histology, the most frequent sources of frustration were communication between patient and physician (19.4%), lack of appropriate information (16.2%), and lack of available/relevant research (14.3%). In contrast, practical sources of frustration were more frequently encountered by older patients (*P* = 0.01; Figure 1C). Older respondents reported lack of appropriate information (14.5%) and distrust of cancer care system (13.9%) as the most frequent sources of frustration.

### Solutions Proposed by Patients

Patients with RCC provided a diverse array of solutions for their identified sources of frustration. Most frequently, as shown in Table 2, it was suggested healthcare providers should demonstrate greater compassion (20.7%). RCC patients also recommended having greater access to informational resources pertaining to diagnostic tests, treatment options and surveillance (15.1%), and conducting further research to identify curative therapies (14.7%).

## Discussion

In the current study, we identify a substantial proportion of patients (71.5%) who have encountered frustration in their care of RCC. To our knowledge, this is the first to use qualitative content analysis to characterize open-ended patient responses related to frustration; the number of valid respondents to this survey (*n* = 450) makes results particularly robust. Emotional causes of frustration were more common than practical causes of frustration. The most frequent drivers of emotional frustration were fear about the risk of disease recurrence or progression. In contrast, the most frequent drivers of practical frustration were lack of information and financial issues. We also sought to obtain suggestions from patients regarding how frustration could be ameliorated. The most frequent response to this query was that health care practitioners should demonstrate more compassion in their interactions with patients.

We found that patients with non-clear cell histology reported a higher rate of both practical and emotional sources of frustration. Non-clear cell histologies represent approximately 15–20% of RCC cases, and include a wide range of diagnoses such as papillary, chromophobe, collecting duct, and medullary (11, 12). With little exception, stage for stage, the prognosis for non-clear cell histologies is worse than that of clear cell disease. The treatment algorithms for advanced non-clear cell histologies often mirror those for clear cell, albeit with modest supporting evidence. Based on our results, the lack of information associated with diagnosis and treatment of non-clear cell histologies may exacerbate frustration for patients as they feel like they have fewer therapeutic options and when offered, treatment for their disease is merely an after-thought as most indications are based on patients with clear cell RCC (one of our so-called “practical” sources of frustration). Providing patient education material developed specifically for rare subtypes could help to diminish fears and open discussions about further treatment options. Highlighting research opportunities for these patients may be another mechanism to address these causes of frustration. For example, several prospective studies are now exploring the role of MET-directed therapies in advanced papillary RCC, given the putative role of MET as a driver of this entity. These include Southwest Oncology Group (SWOG) 1,500, a randomized, phase II study comparing sunitinib to cabozantinib, crizotinib, and savolitinib (13). The SAVOIR study selects patients with papillary RCC upfront with *MET* gene alterations and randomizes to sunitinib or savolitinib (14). Both studies are actively accruing, and patients may take solace in a biology-specific approach to their tumor type.

Females were also noted to have higher rates of frustration derived from both emotional and practical sources. The most common fears amongst females centered on fear of recurrence and progression. Across randomized trials in RCC evaluating systemic agents in the metastatic setting, there appears to be no substantial difference in clinical outcome that can drive this difference. While the prognostic differences may play a slight role in promoting fears pertaining to clinical outcome, there may be underlying psychological differences between males and females that drive this discordance (15, 16).

Beyond gender and histology, which were associated with variable degrees of frustration, it is curious that advanced stage was not associated with increasing frustration. Intuitively, one might assume that patients with metastatic disease would have greater preoccupation with clinical outcome, but our data suggest that frustration is balanced amongst patients with localized and metastatic disease. These data across all stages suggest that many patients struggle with not knowing where they stand and what to do.

Until very recently, no approved adjuvant therapy existed for RCC. For patients with localized disease, this lack of available therapy to prevent recurrence could be a contributing factor for this specific population. With limited treatment options, care paths are unclear for post-nephrectomy patients, and visits with their treating physician consist only of surveillance scans. This could leave patients feeling isolated and abandoned by their care teams. With the availability of adjuvant sunitinib in the US based on the recent phase III S-TRAC trial, multidisciplinary care counseling, including both urologists and medical oncologists is indicated (17). However, the significant toxicity along with modest clinical efficacy could be another source of frustration. Guidelines for surveillance vs. adjuvant treatment vary across entities making follow-up recommendations unclear for patients (and physicians). Patient friendly guidelines and better information about risk of recurrence could help patients make a decision based upon their individual priorities and risk. Additional office visits after localized interventions could open opportunities to assess the emotional status of patients and offer psychological support when warranted, or even, early utilization of supportive oncology clinics to address emotional concerns in the most affected mRCC population. Uniform and structured care plans for patients regardless of stage could provide all patients with additional certainty about the management of their disease.

Limitations of the study include the use of patient-supplied data in the survey, preventing source verification (through the medical record) of data elements such as clinical stage and histology. We also highlight the potential participant bias, in which most of those who answered the survey were probably those seeking support in on-line patient communities. Also, although the intent of the survey was to elicit a broad range of responses with open-ended questions, interpretation of the questions by patients may have been variable. Multiple choice questions might lead to less heterogeneity of responses, and would limit any errors made in our qualitative content analysis. There are also some elements of patient demography which we did not obtain that might be valuable. As one example, we did not ascertain the geographic locations of respondents. Availability of drugs varies widely across different countries, and this may be a major point of frustration. The availability of medical professionals may also be highly variable, resulting in delays in care and challenges in communication with the medical team. The study was also conducted over a relatively narrow time frame, spanning 3 months. With the rapid pace of therapeutic developments in RCC, several key milestones may have been missed. For instance, since June of 2017, several countries have issued approvals for second-line therapies such as nivolumab, cabozantinib and lenvatinib/everolimus (7, 18, 19). Adjuvant sunitinib was approved by the US FDA in November 2017, while EMA in February 2018 did not approve adjuvant sunitinib (8). Finally, it is important to consider that (especially given the likely heterogeneity of the study population) our sample size was relatively small. Thus, our results should be viewed as primarily hypothesis generating.

This study is the first to comprehensively assess sources of frustration amongst patients with RCC. The study highlights key opportunities for reconciling these frustrations through direct suggestions from patients. A dominant theme amongst responses was that health care practitioners should demonstrate more compassion in their clinical encounters. This could be achieved by incorporating psychosocial needs assessments for patients in routine visits. Adding in additional appointments post-diagnosis to ensure that in the patients' physical and emotional well-being is addressed could also be a consideration. Even in resource constrained environments, improvements in this domain should be easily achievable. Other suggestions, such as a lack of information pertaining to diagnostic tests and treatment modalities, could be reconciled by creating a uniform guideline in the RCC community to address the disparities among the various entities (e.g., AUA, EAU, and NCCN). Although educational materials for patients are available on-line and in print, it seems that they do not always address patient questions and concerns. Increased cooperation among physicians and patient advocacy groups could help to identify gaps in existing information and develop more efficient means of distributing materials. Our study highlights that while the pace of therapeutic advances in RCC has been incredibly rapid, there are still many opportunities for us to improve the patient experience through addressing common frustrations.

## Ethics Statement

This study is granted exemption from IRB review, anonymous data were collected using a blind survey.

## Author Contributions

CB, DB, SP, and MS: conception and design; CB, DB, PB, SP, and MS: drafting the manuscript review of the literature; CB, DB, PB, ND, EJ, HH, DG, AB, BL, SP, and MS: critical revision of the manuscript.

### Conflict of Interest Statement

The authors declare that the research was conducted in the absence of any commercial or financial relationships that could be construed as a potential conflict of interest.
