# Supplementary material for: Sources of Frustration Among Patients Diagnosed With Renal Cell Carcinoma
Source: Front Oncol. 2019 Jan 22;9:11. doi: 10.3389/fonc.2019.00011 (PMC6349746; doi:10.3389/fonc.2019.00011)
Supplement: Supplementary file 1 [file Data_Sheet_1.docx]

**Supplemental Table 1.** Survey questions pertaining to adjuvant therapy.

| Questions | Possible Answers |
| --- | --- |
| If taking a drug for one year following surgery could help prevent or delay cancer from recurring, would you: | - Not use it - Use it, if there was moderate toxicity - Use it, only if there was no toxicity - Use it, no matter what toxicity level - Use it, only if it prolongs survival - Don’t know, more information needed - Other |
| If you were able to get treatment to prevent recurrence of your kidney cancer, what would be important for you? | - Insurance coverage - Toxicity of the drug - Increased time to recurrence of cancer - Better surveillance - Physicians recommendation - Available data on efficacy - Longer survival |
